# Zinc finger protein ZBTB20 is an independent prognostic marker and promotes tumor growth of human hepatocellular carcinoma by repressing FoxO1

**DOI:** 10.18632/oncotarget.7425

**Published:** 2016-02-16

**Authors:** Heping Kan, Yuqi Huang, Xianghong Li, Dingli Liu, Jianjia Chen, Miaojiang Shu

**Affiliations:** ^1^ Department of Hepatobiliary Surgery, Nanfang Hospital, Southern Medical University, Guangzhou 510515, China; ^2^ Department of Infectious Disease, Nanfang Hospital, Southern Medical University, Guangzhou 510515, China

**Keywords:** ZBTB20, FoxO1, hepatocellular carcinoma, proliferation, cell cycle

## Abstract

Zinc finger and BTB domain-containing 20 (ZBTB20) is a new BTB/POZ-domain gene and a member of the POK family of transcriptional repressors. Notably, the role of ZBTB20 and its underlying mechanisms involved in hepatocarcinogenesis are poorly investigated. In this study, the expression of ZBTB20 was significantly overexpressed in hepatocellular carcinoma (HCC) tissues. The positive expression of ZBTB20 was associated with large tumor size, high Edmondson-Steiner grading and advanced tumor-node-metastasis (TNM) tumor stage. Additionally, HCC patients with positive expression of ZBTB20 had a poorer 5-year survival. Multivariate analyses revealed that ZBTB20 overexpression was an independent prognostic factor for HCC. Gain- and loss-of-function experiments demonstrated that ZBTB20 promoted HCC cell viability, proliferation, tumorigenicity, and cell cycle progression. Mechanistically, Cyclin D1 and Cyclin E were increased, while p21 and p27 were decreased by ZBTB20 in HCC cells. FoxO1 was inversely correlated with ZBTB20 protein expression in the same cohort of HCC specimens. We further revealed that FoxO1 was transcriptionally repressed by ZBTB20 in HCC. Moreover, restoration of FoxO1 expression partially abrogated ZBTB20-induced HCC cell proliferation and growth entry *in vitro* and *in vivo*. Collectively, these results indicate that ZBTB20 may serve as a prognostic marker and promotes tumor growth of HCC via transcriptionally repressing FoxO1.

## INTRODUCTION

Hepatocellular carcinoma (HCC) is the fifth most common malignancy and the third cause of cancer-related death worldwide [1]. Although various treatments including hepatectomy and transcatheter arterial chemoembolization are applied in clinical practice, the prognosis of HCC is still poor. Multiple studies have shown that HCC results from dysregulation of oncogenes and/or tumor suppressors [2]. However, the molecular mechanisms involved in the initiation and progression of HCC remain poorly understood. Therefore, identification of critical carcinogenic mechanisms will contribute to discovery of novel therapeutic targets for HCC.

Zinc finger and BTB domain-containing 20 (ZBTB20), also named HOF [3], DPZF [4], and ZNF288 [5], containing an intact N-terminal BTB domain and a C-terminal zinc finger domain, is a new member of the POK (POZ and Krüppel) family of transcriptional repressors [6, 7]. ZBTB20 participate in various cellular functions including transcriptional regulation, cellular proliferation, tumorigenesis, ion channel assembly, and chromatin remodeling [8–10]. A subset BTB/POZ protein has been implicated in human cancer, such as BCL-6, PLZF, and NAC-1. The BCL-6 and PLZF proteins are involved in several types of B-cell lymphomas and acute promyelocytic leukemia [11–13]. NAC-1 is significantly overexpressed in ovarian serous carcinoma and predicts early recurrence [14]. For ZBTB20, initially studies demonstrated that ZBTB20 plays an essential role in the specification of CA1 field identify in the developing hippocampus [15]. Besides, ZBTB20 regulates β cell function and glucose homeostasis in mice model [16]. Increasing studies have disclosed the aberrant expression and role of ZBTB20 in carcinogenesis. ZBTB20 was increased in non-small cell lung cancer and promotes cell proliferation [17]. In mouse, ZBTB20 is developmentally up-regulated in postnatal liver and acts as a key transcription repressor of AFP [7]. Furthermore, Wang et al. reported that increased expression of ZBTB20 was associated with poor prognosis in HCC patients [18]. However, Weng et al. demonstrated that ZBTB20 was down-regulated following 70% hepatectomy in mice, suggesting that ZBTB20 may inhibit cell proliferation in the hepatocytes [19]. Therefore, the role of ZBTB20 in HCC progression remains uncharacterized.

Recent studies indicate that FoxOs regulate cell physiological process by modulating genes involved in apoptosis, cell cycle transitions, apoptosis, angiogenesis, stress resistance, as well as cell differentiation and glucose metabolism [20–22]. Dysregulation of FoxO1 were implicated in liver fibrogenesis and HCC progression [23, 24]. In the current study, overexpressed ZBTB20 protein was associated with adverse clinicopathologic features and poor prognosis of HCC patients. ZBTB20 promoted HCC cell viability, proliferation, tumorigenicity and cell cycle progression by directly suppressing FoxO1, and subsequently up-regulated Cyclin D1/Cyclin E and down-regulated p21/p27.

## RESULTS

### Clinical significance of ZBTB20 in HCC specimens

**Figure 1:**
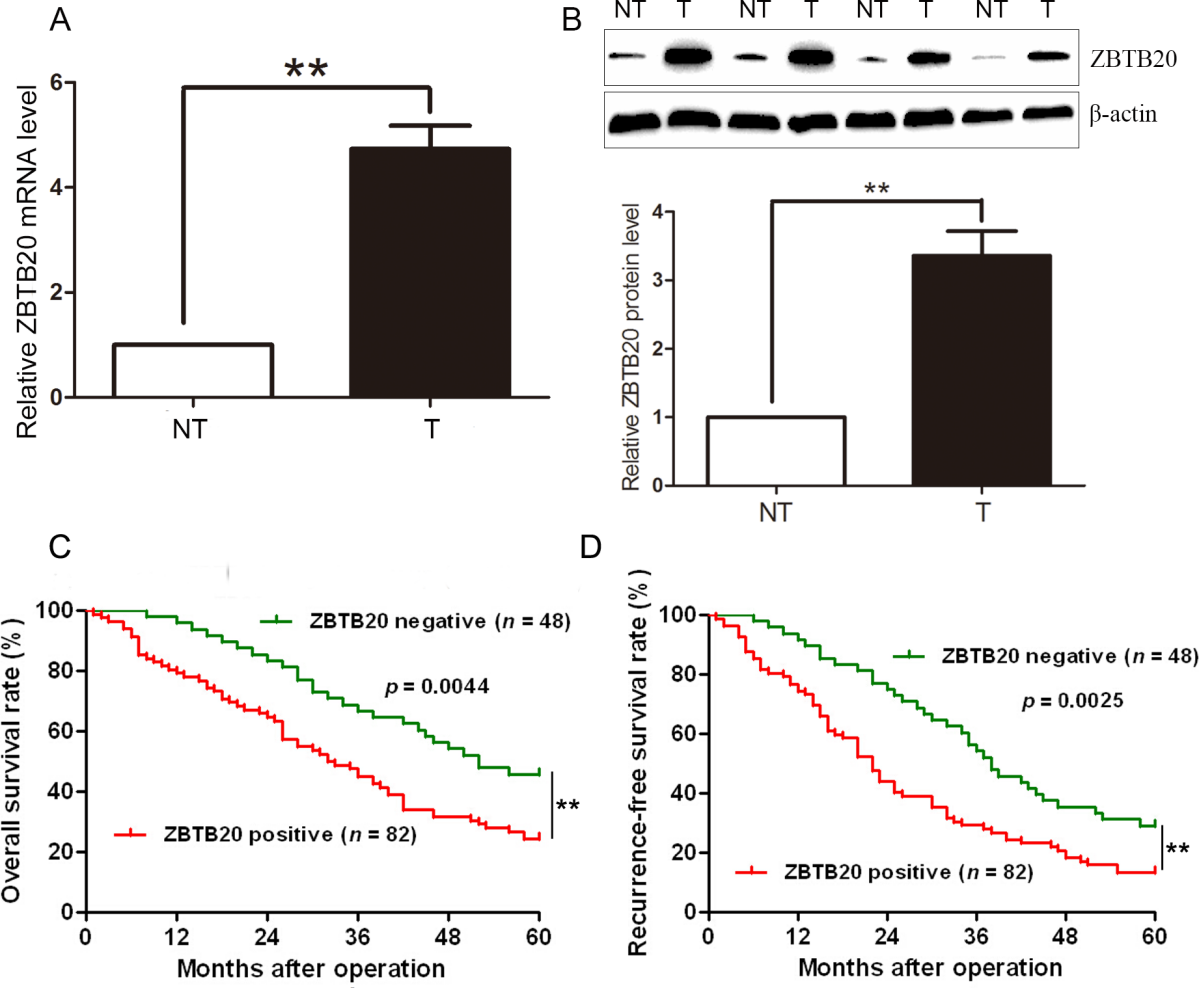
Expression of ZBTB20 and its clinical significance in HCC cases (**A**) The expression of ZBTB20 mRNA in tumor (T) was significantly higher than that in matched nontumor tissues (NT). *n* = 40, **P < 0.01 by *t* test. (**B**) Representative Western blots analysis of ZBTB20 expression in HCC and matched tumor-adjacent tissues was shown. Quantitative data indicated that ZBTB20 protein was expressed at a significant higher level in HCC as compared with noncancerous tissues. *n* = 40, **P < 0.01 by *t* test. (**C**) Kaplan-Meier 5-year overall and (**D**) recurrence-free survival curves for HCC patients according to their ZBTB20 protein expression status. 130 HCC patients were divided into ZBTB20 positive group (*n* = 82) and negative group (*n* = 48) according to IHC scores. **P < 0.01 by log-rank test.

We tested the expression of ZBTB20 protein by immunohistochemistry in 130 pairs of HCC and matched noncancerous tissue samples. ZBTB immunoreactivity was considered as either negative or positive according to immunohistochemistry (IHC) scores. Positive expression of ZBTB20 was detected in 82 (63.1%) of the HCC specimens, whereas only 42 (32.3%) of the normal tumor-adjacent tissues showed a positive ZBTB20 signal (*P* < 0.01). Furthermore, 40 pairs of samples were randomly selected and subjected to qRT-PCR and Western blot. We found that the levels of ZBTB20 mRNA and protein in HCC tissues were significantly higher than those in matched normal tumor-adjacent tissues (*P* < 0.01, Figure 1A and 1B). As shown in Table 1, clinical association analysis using a Pearson chi-squared test revealed that the expressions of ZBTB20 were evidently higher in HCC patients with large tumor size (*P* = 0.010), high Edmondson-Steiner grading (*P* = 0.042) and advanced TNM tumor stage (*P* = 0.010).

**Table 1:** Correlation between the clinicopathologic characteristics and ZBTB20 expression in HCC

Characteristics		Total No. of patients, *n* = 130	No. of patients		*P*
			ZBTB20 positive	ZBTB20 negative	
Age (y)	< 50	54	34	20	0.982
	≥ 50	76	48	28	
Sex	Male	98	64	34	0.357
	Female	32	18	14	
HBV	Absent	42	27	15	0.844
	Present	88	55	33	
Serum AFP level (ng/mL)	< 20	28	18	10	0.881
	≥ 20	102	64	38	
Tumor size (cm)	< 5	49	24	25	0.010*
	≥ 5	81	58	23	
No. of tumor nodules	1	102	63	39	0.554
	≥ 2	28	19	9	
Cirrhosis	Absent	54	32	22	0.447
	Present	76	50	26	
Venous infiltration	Absent	96	62	34	0.550
	Present	34	20	14	
Edmondson-Steiner grading	I + II	98	57	41	0.042*
	III + IV	32	25	7	
TNM tumor stage	I + II	97	55	42	0.010*
	III + IV	33	27	6	

HCC, hepatocellular carcinoma; HBV, hepatitis B virus; AFP, alpha-fetoprotein; TNM, tumor-node-metastasis.

* Statistically significant.

### Increased expression of ZBTB20 correlates with a poorer 5-year survival for HCC patients

A total of 130 HCC patients with complete clinical information were included to disclose the prognostic significance of ZBTB20 in HCC. Our data indicated that 5-year overall survival in ZBTB20 positive expression group (*n* = 82) was 24.39%, as compared with 45.83% in negative expression group (*n* = 48). Statistic analyses showed that HCC patients in ZBTB20 positive expression group had a significant poorer 5-year survival (log-rank = 8.131, *P* = 0.0044; Figure 1C). The median recurrence-free survival times in ZBTB20 positive and negative expression group were 22.0 and 38.0 months, respectively. Kaplan-Meier analysis also revealed that positive expression of ZBTB20 was associated with a shorter recurrence-free survival time (log-rank = 9.158, *P* = 0.0025; Figure 1D). These data suggest that ZBTB20 may function as a potential prognostic marker in HCC. Furthermore, Multivariate Cox regression analysis explored that ZBTB20 overexpression was an independent factor for indicating both 5-year overall and recurrence-free survival of HCC patients (*P* = 0.008 and 0.038, respectively; Table 2).

**Table 2:** Multivariate Cox regression analysis of 5-year OS and RFS of 130 HCC patients

Variables	OS			RFS		
	HR	95% CI	*P*	HR	95% CI	*P*
Tumor size (cm) (< 5 vs ≥ 5)	1.092	0.500–2.386	0.824	1.670	0.787–3.545	0.182
Edmondson-Steiner grading (I + II vs III + IV)	3.233	1.654–4.265	0.013*	1.971	0.827–4.696	0.126
TNM tumor stage (I + II vs III + IV)	2.989	1.678–3.476	0.015*	2.034	1.398–2.573	0.005*
ZBTB20 expression (negative vs positive)	2.652	1.459–3.188	0.008*	4.123	2.456–5.033	0.038*

OS, overall survival; RFS, recurrence-free survival; TNM, tumor-node-metastasis; HR, hazard ratio; CI, confidence interval.

* Statistically significant.

### ZBTB20 promotes HCC cell proliferation *in vitro*

**Figure 2:**
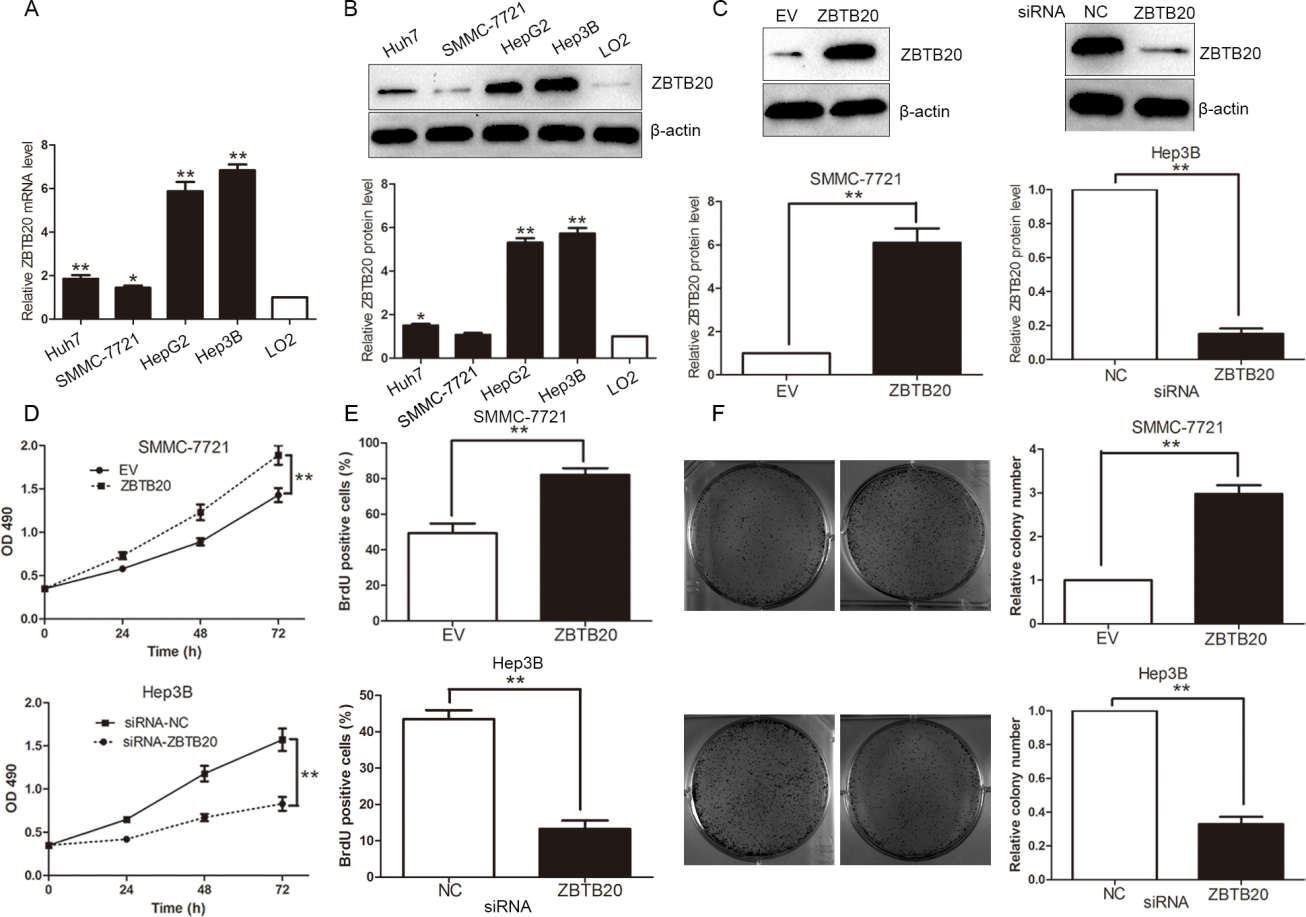
ZBTB20 facilitates proliferation and tumorigenicity of HCC cells (**A** and **B**) Comparing differences in the expression levels of ZBTB20 mRNA and protein between HCC cell lines with different proliferative potentials and the immortalized hepatic cell line. *n* = three repeats with similar results, *P < 0.05 and **P < 0.01 by ANOVA. (**C**) SMMC-7721 cells that were tranfected with empty vector (EV) or ZBTB20 retroviruses were subjected to immunoblotting for ZBTB20. ZBTB20 was knocked down by a specific siRNA and confirmed by Western blot in Hep3B cells. *n* = three repeats with similar results, **P < 0.01 by *t* test. (**D**) As assessed by MTT assays, ZBTB20 overexpression enhanced cell viability of SMMC-7721 cells and ZBTB20 knockdown was found to reduce Hep3B cell viability. *n* = three repeats with similar results, **P < 0.01 by ANOVA. (**E**) Cell proliferation as measured by BrdU incorporation assays was promoted by ZBTB20 overexpression in SMMC-7721 cells and suppressed by ZBTB20 knockdown in Hep3B cells. *n* = three repeats with similar results, **P < 0.01 by *t* test. (**F**) Representative colony formation assays were shown in HCC cells with altered ZBTB20 expression. Quantitative data disclosed that the ability of colony formation was enhanced after ZBTB20 overexpression in SMMC-7721 cells and reduced after ZBTB20 knockdown in Hep3B cells. *n* = three repeats with similar results, **P < 0.01 by *t* test.

To investigate the role of ZBTB20 in HCC cells, we first evaluated the levels of ZBTB20 mRNA and protein in a human normal liver cell line (LO2) and HCC cell lines (Hep3B, Huh7, HepG2 and SMMC-7721). The levels of ZBTB20 mRNA and protein were up-regulated in HCC cell lines as compared with LO2 (*P* < 0.05, Figure 2A and 2B). As SMMC-7721 cell line showed the lowest basal expression of ZBTB20 in four HCC cell lines, we enforced ZBTB20 expression in SMMC-7721 cells employing retroviruses-mediated empty vector (EV) or ZBTB20 (*P* < 0.01, Figure 2C). Otherwise, a specific siRNA was used to knock down the endogenous ZBTB20 in Hep3B cells (*P* < 0.05, Figure 2C), which has higher basal expression of ZBTB20 than other three HCC cell lines. MTT and BrdU incorporation assays were performed to test the effect of altering ZBTB20 levels on tumor cell viability and proliferation, respectively. As expected, ZBTB20 overexpression promoted the viability and proliferation of SMMC-7721 cells, while ZBTB20 knockdown inhibited cell viability and proliferation in Hep3B cells (*P* < 0.01, Figure 2D and 2E). Colony formation assays showed that ZBTB20 overexpression promoted and ZBTB20 silencing inhibited the colony formation capacity of HCC cells (*P* < 0.01, Figure 2F).

### ZBTB20 affects expression of the cell-cycle regulators

**Figure 3:**
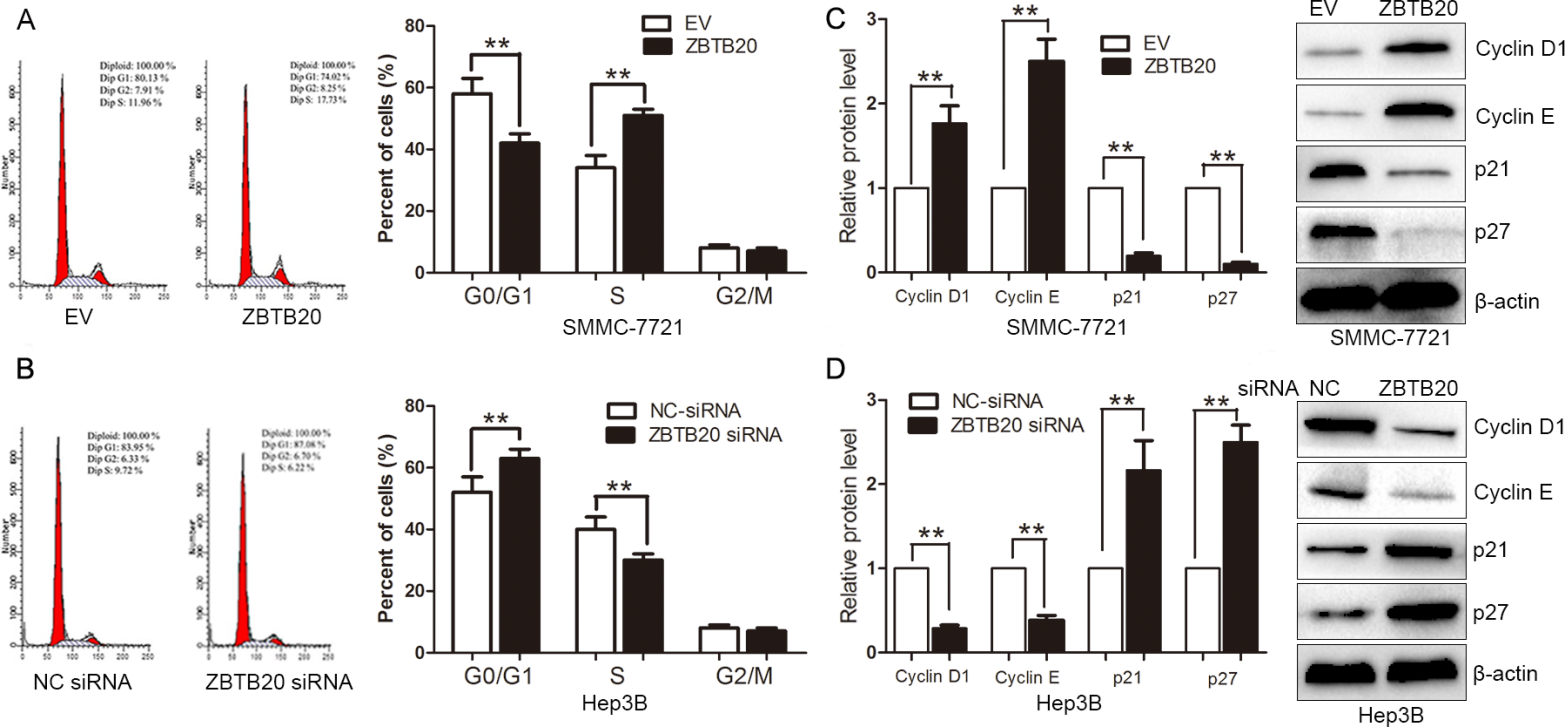
ZBTB20 promotes cell cycle progression in HCC cells (**A**) SMMC-7721 cells that were transfected with empty vector (EV) or ZBTB20 were subjected to flow cytometry for cell cycle. ZBTB20 overexpression promoted cell cycle progression with decrease of cells in G0/G1 phase and increase of cells in S phase. *n* = three repeats with similar results, **P < 0.01 by *t* test. (**B**) Cell cycle as measured by flow cytometry after ZBTB20 knockdown showed an increase of Hep3B cells in G0/G1 phase and decrease of cells in S phase. *n* = three repeats with similar results, **P < 0.01 by *t* test. (**C**) ZBTB20 overexpression up-regulated the expressions of Cyclin D1 and Cyclin E, and down-regulated the levels of p21 and p27 in SMMC-7721 cells. *n* = three repeats with similar results, **P < 0.01 by *t* test. (**D**) Hep3B cells that were transfected with non-targeting (NT) or ZBTB20 siRNA were subjected to Western blot for Cyclin D1, Cyclin E, p21 and p27. *n* = three repeats with similar results, **P < 0.01 by *t* test.

Next, we examined whether ZBTB20 regulated cell-cycle progression in HCC cells. As determined by flow cytometry, ZBTB20 overexpression significantly reduced the percentage of cells in G1/G0 phase and increased the percentage of cells in S phage (*P* < 0.01, Figure 3A). Cyclin D1 and Cyclin E are involved in promoting cell-cycle progression, while p21 and p27 are known as cyclin-dependent kinase inhibitors. Here, we found that the expressions of Cyclin D1 and Cyclin E were up-regulated, and the levels of p21 and p27 were down-regulated in ZBTB20 overexpressing SMMC-7721 cells (*P* < 0.01, Figure 3B). Furthermore, ZBTB20 knockdown increased the percentage of cells in G1/G0 phase and reduced the percentage of cells in S phase (*P* < 0.01, Figure 3C). Western blot analyses indicated that Cyclin D1 and Cyclin E were down-regulated, and the expression of p21 and p27 were up-regulated after ZBTB20 knockdown in Hep3B cells (*P* < 0.01, Figure 3D). Taken together, these results suggest that ZBTB20 plays an oncogenic role in HCC by promoting cell viability, proliferation, tumorigenicity and cell cycle progression.

### ZBTB20 inversely regulates FoxO1 abundance in HCC

**Figure 4:**
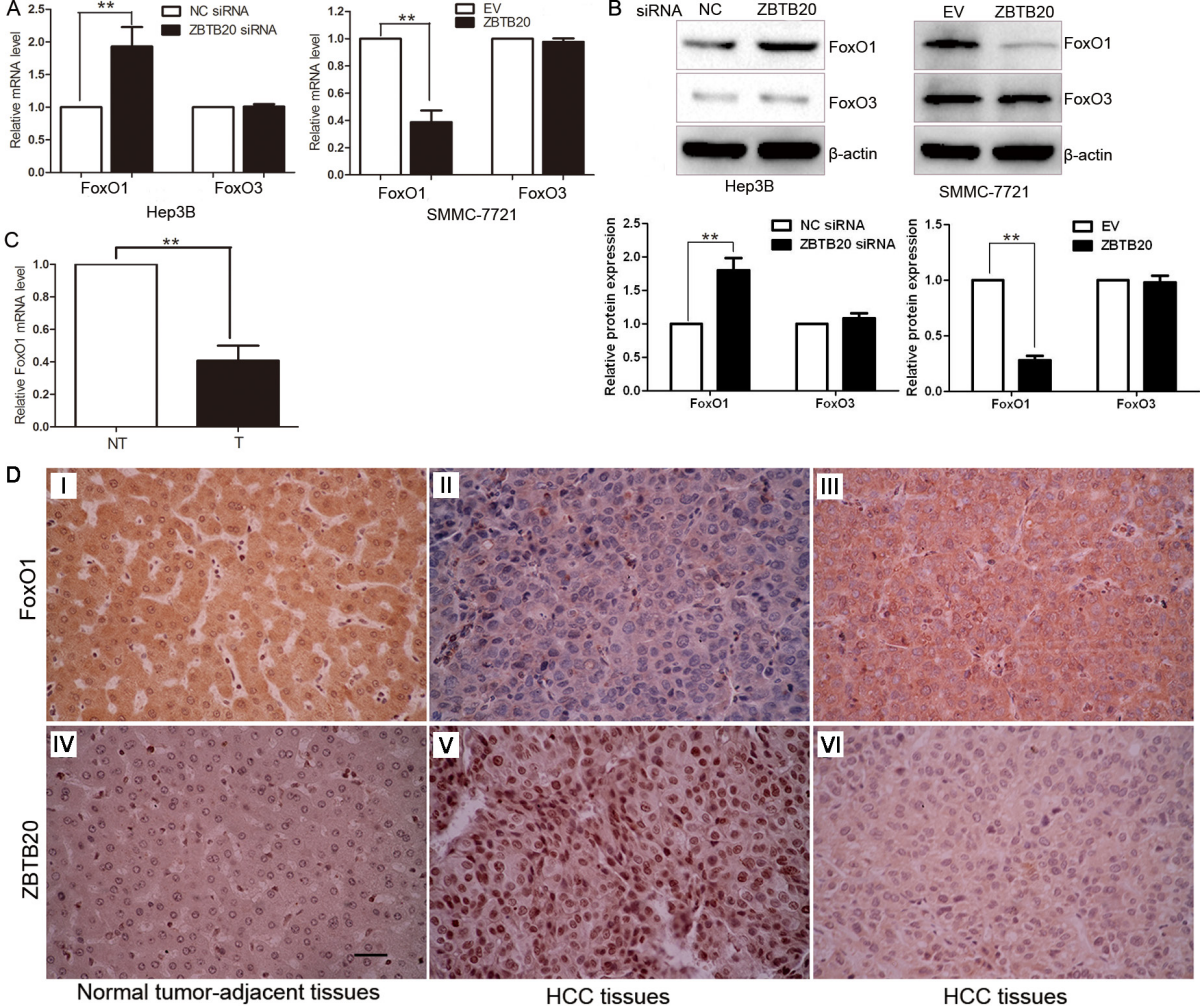
ZBTB20 inversely regulates FoxO1 abundance in HCC (**A**) ZBTB20 knockdown up-regulated the level of FoxO1 mRNA in Hep3B cells, and the expression of FoxO1 mRNA was reduced after ZBTB20 overexpression in SMMC-7721 cells. However, the expression of FoxO3 did not changed after ZBTB20 alteration. *n* = three repeats with similar results, **P < 0.01 by *t* test. (**B**) Hep3B and SMMC-7721 cells that were transfected with ZBTB20 siRNA and ZBTB20 retroviruses, respectively, and subjected to immunoblotting for FoxO1 and FoxO3. *n* = three repeats with similar results, **P < 0.01 by *t* test. (**C**) The expression of FoxO1 mRNA in tumor (T) was significantly lower than that in matched nontumor tissues (NT). *n* = 40, **P < 0.01 by *t* test. (**D**) In cases of high ZBTB20 protein expression (V), there was no detectable FoxO1 protein expression (II) in the same tissue section. In contrast, in the case of low ZBTB20 protein expression (IV, VI), there was strong FoxO1 protein expression (I, III). Scale bar: 20 μm.

Previous studies have shown that the anti-tumor activity of FoxOs results from their pro-apoptotic and cell cycle inhibitory effects [25, 26]. Among them, FoxO1 transcriptionally regulates genes related to cell cycle progression, including p21, p27 and Cyclin D1 [26, 27]. Moreover, FoxO1 shows the most abundance in insulin-responsive tissues (e.g., liver) and its dysregulation has been found in several human cancers [28]. ZBTB20 knockdown increased the levels of FoxO1 mRNA and protein in Hep3B cells (*P* < 0.01, Figure 4A and 4B). Moreover, FoxO1 mRNA and protein were significantly down-regulated by ZBTB20 overexpression in SMMC- 7721 cells (*P* < 0.01, Figure 4A and 4B). In addition, the expression of FoxO3, another member of FoxO transcription factors, was not affected by ZBTB20 alteration (Figure 4A and 4B). 40 samples of HCC and matched tumor-adjacent tissues were randomly selected and subjected to qRT-PCR for FoxO1 mRNA. The levels of FoxO1 mRNA were significantly decreased in HCC tissues as compared with matched nontumor tissues (*P* < 0.01, Figure 4C). Moreover, we examined the correlation between ZBTB20 and FoxO1 expression in serial sections of HCC cases by immunohistochemical staining. The expression of FoxO1 protein in HCC tissues was significantly lower than those in matched noncancerous tissues [40.0% (52/130) *vs* 58.5 (76/130); *P* < 0.01]. Furthermore, We found a strong inverse correlation between ZBTB20 and FoxO1 expression in HCC tissues (*r* = −0.481; *P* < 0.001; Figure 4D).

### FoxO1 gene promoter is a direct transcriptional target of ZBTB20

**Figure 5:**
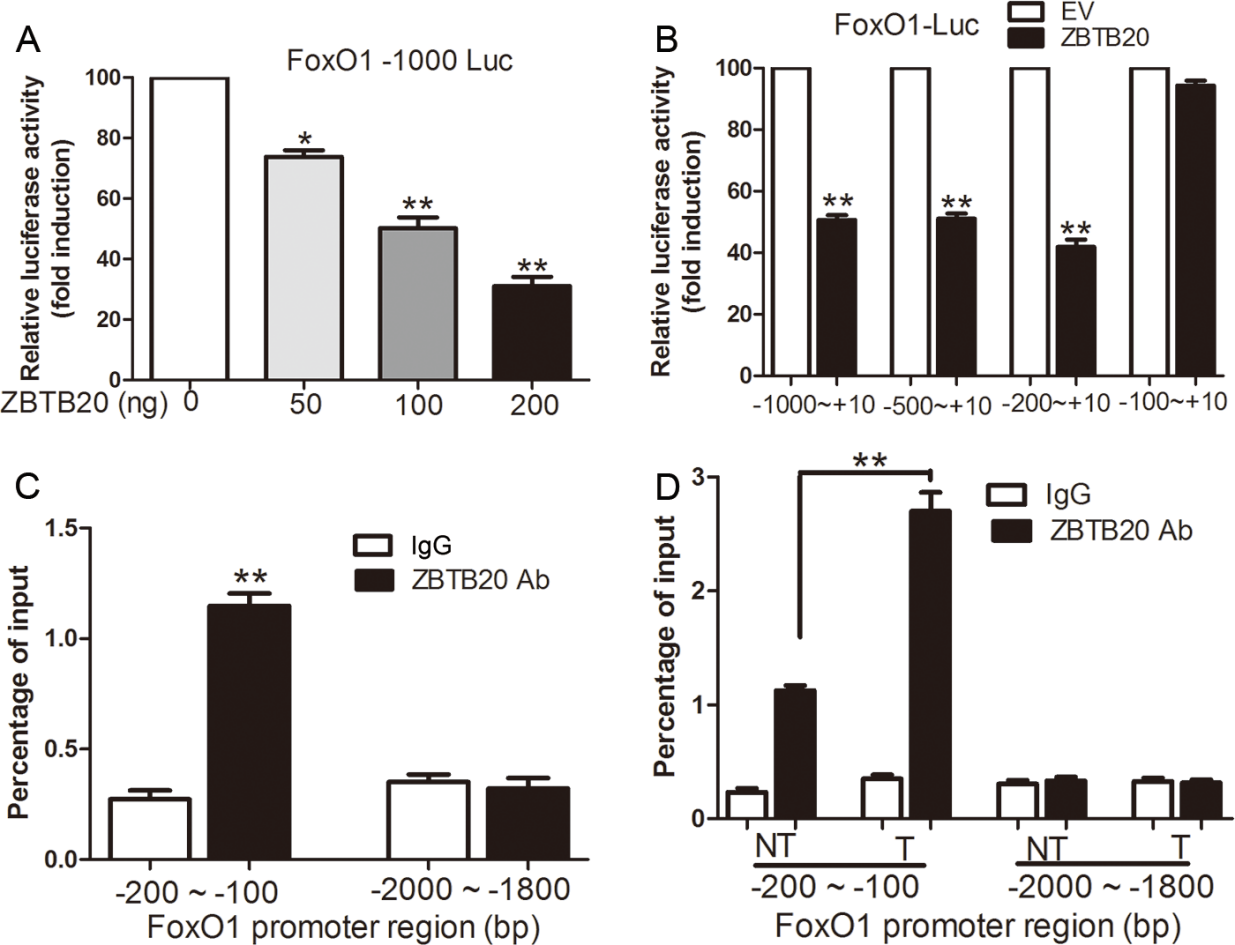
ZBTB20 transcriptionally represses FoxO1 promoter activity (**A**) Construction and luciferase reporter analysis of human FoxO1 promoter. The promoter region from −1000 to +10 bp was cloned and co-transfected with ZBTB20 expression plasmids in SMMC-7721 cells. The transcription start site was set as +1 bp. *n* = three repeats with similar results, *P < 0.05 and **P < 0.01 by ANOVA. (**B**) Transcriptional activity of human FoxO1 promoter by series of deletion. SMMC-7721 cells were transfected with the indicated plasmids. *n* = three independent experiments, **P < 0.01 by *t* test. (**C**) ChIP assays to show the recruitment of ZBTB20 onto FoxO1 promoter. The promoter region from −2000 to −1800 bp was set as a negative control. Real-time PCR was performed to quantify the binding. SMMC-7721 cells were cultured for 48 h after seeding and then subjected to ChIP assays. *n* = three independent experiments, **P < 0.01 by *t* test. (**D**) ChIP analysis of ZBTB20 binding to the FoxO1 promoter in cancer (T) and matched noncancerous tissue (NT). *n* = six, **P < 0.01 by *t* test.

To further explore the underlying mechanisms of ZBTB20 involved in FoxO1 repression, the promoter regions of FoxO1 gene from −1000 bp to +10 bp were cloned and inserted into luciferase reporter vectors. Importantly, ZBTB20 repressed the reporter FoxO1 −1000 Luc in a dose-dependent manner, which was consistent with the down-regulation of FoxO1 (*P* < 0.05, Figure 5A). Next, we performed a serial deletion of this promoter to further identify the potential region, which was targeted by ZBTB20 for transcriptional suppression of FoxO1. Herein, we found that deletion of −200 bp to −100 bp regions largely impaired the inhibitory effects of ZBTB20 on the FoxO1 reporters, indicating that the ZBTB20-reponsive element may be located in the −200 bp to −100 bp region of FoxO1 promoter (*P* < 0.01, Figure 5B). In addition, chromatin immunoprecipitation assays also confirmed that ZBTB20 was able to bind with this region in SMMC-7721 cells (*P* < 0.01, Figure 5C). Notably, this association of ZBTB20 with the FoxO1 promoter was substantially increased in HCC tissues (*P* < 0.01, Figure 5D).

### ZBTB20 promotes proliferation and cell cycle progression through suppressing FoxO1 in HCC

**Figure 6:**
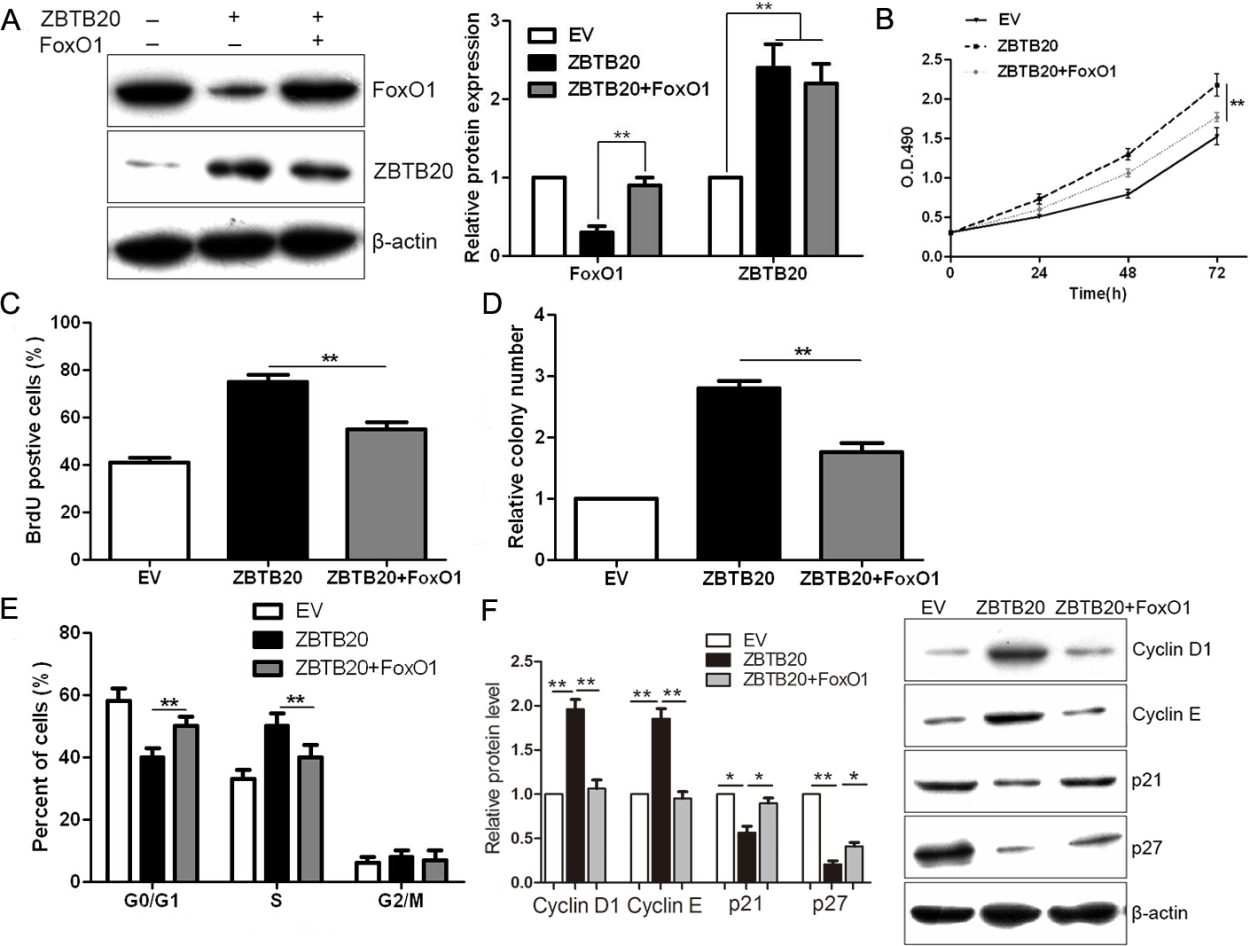
ZBTB20's promotion of cancer cell growth is abolished by FoxO1 (**A**) Western blot analysis indicated that FoxO1 was successfully restored by retroviruses in ZBTB20 transfected SMMC-7721 cells. *n* = three repeats with similar results, **P < 0.01 by ANOVA. (**B**) As assessed by MTT assays, FoxO1 reduced cell viability in ZBTB20 overexpressing SMMC-7721 cells. *n* = three repeats with similar results, **P < 0.01 by ANOVA. (**C**) ZBTB20 overexpressing SMMC-7721 cell proliferation as measured by BrdU incorporation assays was inhibited by FoxO1. *n* = three repeats with similar results, **P < 0.01 by ANOVA. (**D**) The ability of colony formation was reduced after FoxO1 restoration in ZBTB20 overexpressing SMMC-7721 cells. *n* = three repeats with similar results, **P < 0.01 by ANOVA. (**E**) FoxO1 inhibited cell cycle of ZBTB20 overexpressing SMMC-7721 cell with increase of cells in G0/G1 phase and decease of cells in S phase. *n* = three repeats with similar results, **P < 0.01 by ANOVA. (**F**) FoxO1 down-regulated the expressions of Cyclin D1 and Cyclin E, and up-regulated the levels of p21 and p27 in ZBTB20 overexpressing SMMC-7721 cells. *n* = three repeats with similar results, **P < 0.01 by ANOVA.

**Figure 7:**
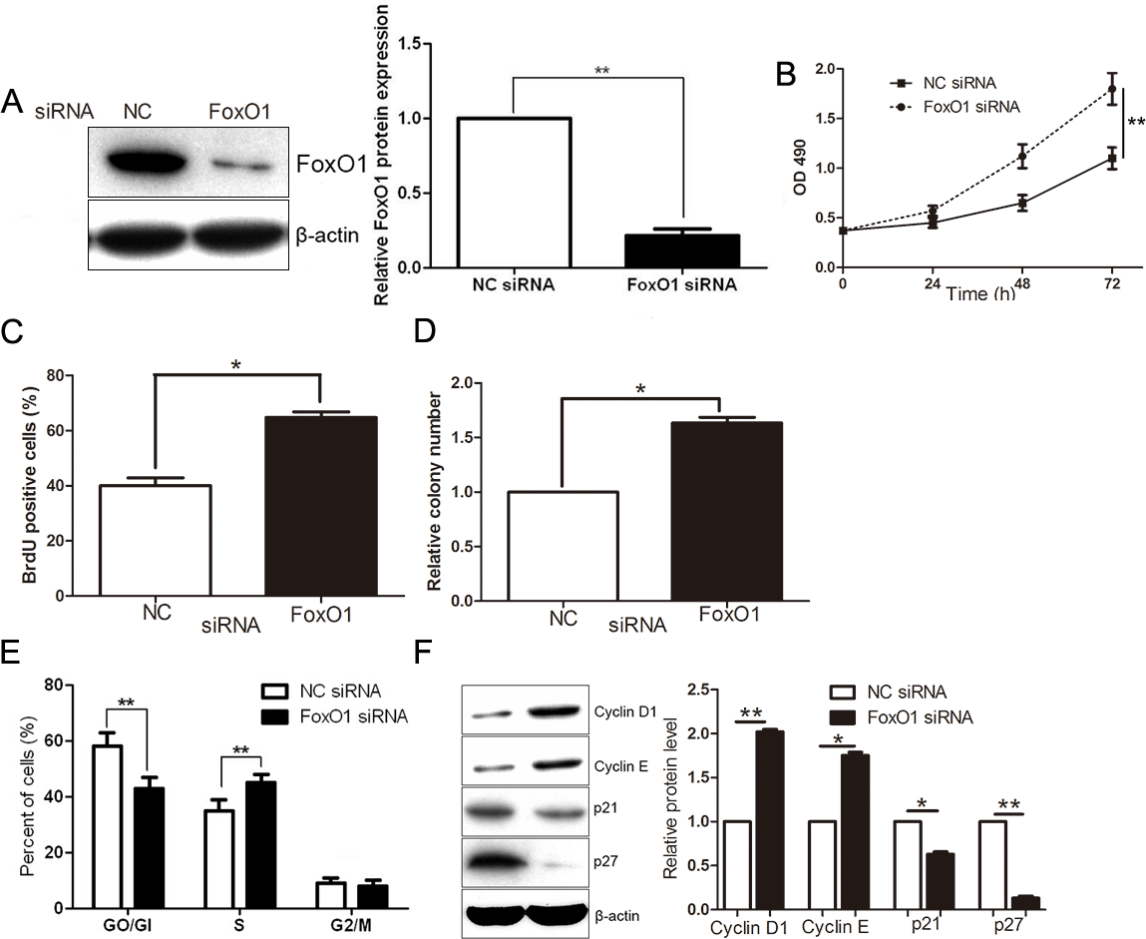
FoxO1 knockdown inhibits cancer cell growth (**A**) SMMC-7721 cells that were transfected with non-targeting (NT) or FoxO1 siRNA were subjected to Western blot for FoxO1. *n* = three repeats with similar results, **P < 0.01 by *t* test. (**B**) As assessed by MTT assays, FoxO1 knockdown enhanced cell viability in SMMC-7721 cells. *n* = three repeats with similar results, **P < 0.01 by ANOVA. (**C**) SMMC-7721 cell proliferation as measured by BrdU incorporation assays was promoted by FoxO1 knockdown. *n* = three repeats with similar results, *P < 0.05 by *t* test. (**D**) The ability of colony formation was increased after FoxO1 knockdown in SMMC-7721 cells. *n* = three repeats with similar results, *P < 0.05 by *t* test. (**E**) FoxO1 knockdown promoted cell cycle of SMMC-7721 cell with decrease of cells in G0/G1 phase and increase of cells in S phase. *n* = three repeats with similar results, **P < 0.01 by *t* test. (**F**) FoxO1 knockdown up-regulated the expressions of Cyclin D1 and Cyclin E, and down-regulated the levels of p21 and p27 in SMMC-7721 cells. *n* = three repeats with similar results, *P < 0.05 and **P < 0.01 by *t* test.

To determine whether the FoxO1 protein participates in ZBTB20-mediated cell proliferation and cell-cycle progression in HCC, FoxO1 was subsequently up-regulated in ZBTB20 overexpressing SMMC-7721 cells via plasmid transfection (*P* < 0.01, Figure 6A). Restoration of FoxO1 expression partially abrogated the effects of ZBTB20 overexpression on SMMC-7721 cells, leading to a significant reduction in cell viability, proliferation, tumorigenicity and cell cycle progression (*P* < 0.01, Figure 6B–6E). In addition, we found that FoxO1 overexpression resulted in an increase of p21 and p27, and decrease of Cyclin D1 and Cyclin E (*P* < 0.05, Figure 6F). Moreover, a specific siRNA was used for endogenous FoxO1 silencing in SMMC-7721 cells (*P* < 0.01, Figure 7A). As expected, FoxO1 knockdown resulted in an increase of cell viability, proliferation, tumorigenicity and cell cycle progression (*P* < 0.05, Figure 7B–7E). Meanwhile, FoxO1 knockdown up-regulated Cyclin D1 and Cyclin E, and down-regulated p21 and p27 in SMMC-7721 cells (*P* < 0.05, Figure 7F).

**Figure 8:**
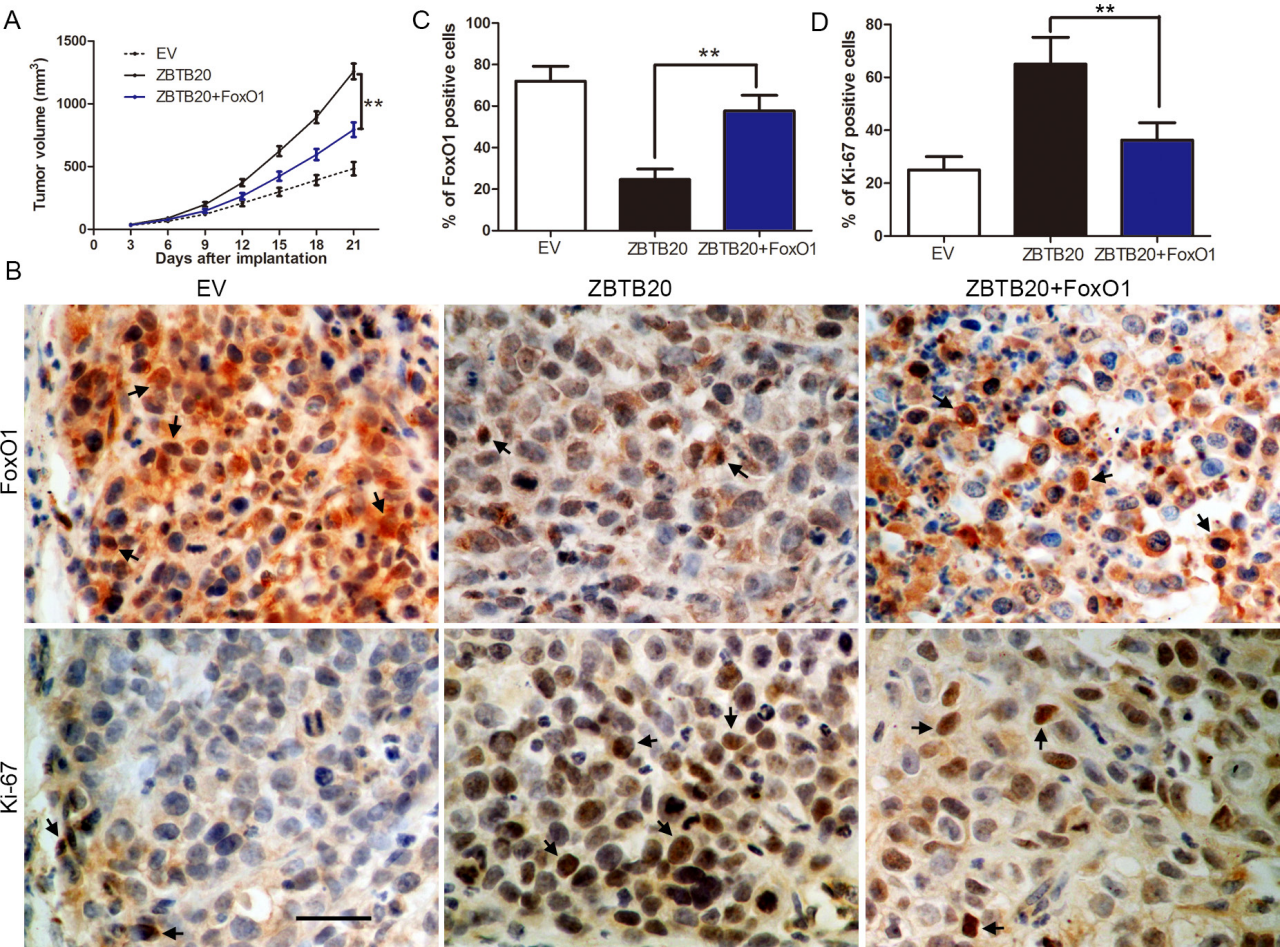
FoxO1 partially abrogates ZBTB20's promotion of tumor growth (**A**) Control SMMC-7721 cells (empty vector, EV, *n* = six), ZBTB20 overexpressing SMMC-7721 cells (ZBTB20, *n* = six) and co-expressing SMMC-7721 cells (ZBTB20 + FoxO1, *n* = six), respectively, were implanted into nude mice via subcutaneous injection. ZBTB20 overexpressing SMMC-7721 cells exhibited a greater tumor-promotive effect compared with control cells. However, restoration of FoxO1 expression inhibited tumor growth, compared with the ZBTB20 group. **P < 0.01 by ANOVA. (**B**) Tumor nodules were subjected to immunohistochemical staining for FoxO1 and Ki- 67. (**C** and **D**) Representative immunostaining revealed that ZBTB20 overexpression significantly reduced the number of FoxO1 positive cells and increased the number of Ki-67 staining cells. However, the percentage of FoxO1 positive cells in tumors arising from the ZBTB20 + FoxO1 group was significantly higher than that in the tumors from the ZBTB20 group. And the percentage of Ki-67 staining cells in the ZBTB20 + FoxO1 group was significantly lower than that in the ZBTB20 group. *n* = six, **P < 0.01 by ANOVA. Scale bar: 20 μm. Black arrowheads indicated the positive cells.

Next, SMMC-7721 cells that were infected with different retroviruses were implanted into nude mice via subcutaneous injection. Tumor growth cures, generated over 21 days, revealed that ZBTB20 overexpression promoted tumor growth of SMMC-7721 cells in mice. Restoration of FoxO1 expression partially slowed down tumor growth of ZBTB20 overexpressing SMMC-7721 cells (*P* < 0.01, Figure 8A). We performed immunohistochemistry for FoxO1 and Ki-67 in the xenografted tissues. As expected, ZBTB20 overexpression down-regulated FoxO1 protein and promoted proliferation *in vivo*. FoxO1 partially abolished the promotive effects of ZBTB20 on HCC growth with a significant less number of Ki-67 positive staining cells (*P* < 0.01, Figure 8B–8D). Taken together, these data indicate that FoxO1 may function as a downstream factor in ZBTB20-induced proliferation and cell cycle progression in HCC.

## DISCUSSION

Hepatocellular carcinoma (HCC) is a complex disease caused by a combination of both genetic and environmental factors [29]. However, the underlying mechanisms of HCC have not yet been illustrated. In the present study, our data showed that increased expression of ZBTB20 was obviously correlated with large tumor size, high Edmondson-Steiner grading and advanced TNM tumor stage in HCC. In the previous research, ZBTB20 was considered as a repressor of AFP in liver [7]. However, no correlation between serum AFP level and HCC tissue ZBTB20 protein expression was found in our study, which was consistent with prior study [18]. Considering the possible discrepancy of serum and liver tissue AFP level, Wang et al. showed that the ZBTB20 level was negatively correlated with the staining degree of AFP in HCCs, while the serum AFP level had no significant correlation with the tissue AFP [18]. These may be a possible explanation for our observation. Furthermore, our data indicated that ZBTB20 overexpression was an independent factor for predicting reduced survival of HCC patients. Thus, These results demonstrate that ZBTB20 is critical for prognosis determination in HCC patients.

Next, ZBTB20 was found to be overexpressed in HCC cell lines. Gain- and loss-of function studies indicated that ZBTB20 promoted HCC cell viability, proliferation, tumorigenicity and cell cycle progression. Additionally, the abundance of Cyclin D1, Cyclin E, p21 and p27, key regulators of cell cycle progression, were up- or down-regulated by ZBTB20 in HCC cells. As a general tumor suppressor, previous studies have shown that FoxO transcription factors function as important tumor suppressors [30]. Additionally, recent studies have indicated that aberrant expression of FoxO genes is associated with the development of human tumors [31], such as HCC [32]. FoxO1 is a potent transcriptional activator that triggers the expression of genes involved in cell cycle arrest, apoptosis and hypoxia responsiveness [24]. We confirmed that ZBTB20 inversely regulated FoxO1 but not FoxO3 abundance in HCC cells. Furthermore, FoxO1 was inversely associated with ZBTB20 protein expression in HCC tissues. Importantly, we demonstrated that ZBTB20 repressed FoxO1 expression through directly binding to its proximal promoter region, indicating a critical role of ZBTB20 in the transcriptional regulation of FoxO1. Thus, the most likely scenario is hypothesized that ZBTB20 interferes with transcriptional stimulation by activators or recruits corepressors to inhibit transcription and remodel chromatin structure. It will be worthwhile to examine whether there is synergy between these distinct repression mechanisms in further study. The effects of ZBTB20 overexpression on cell viability, proliferation, tumorigenicity and cell cycle progression were reverted by retrovirus-mediated FoxO1 overexpression *in vitro* and *in vivo*. ZBTB20 targets various oncoproteins that play important roles in regulating proliferation and cell cycle [16, 17, 33, 34]. Nevertheless, ZBTB20 mediated FoxO1 inhibition might account for growth entry and cell cycle progression in HCC.

Data linking ZBTB20 and HCC is limited. Wang et al. reported that elevated expression of ZBTB20 was associated with adverse clinicopathological features and poor prognosis of HCC patients [18]. Our results further confirmed the above findings. These data from two different research centers will make the conclusion more convincing. Notably, the role of ZBTB20 is pleiotropic. Sutherland et al. reported that ZBTB20 null mice exhibited a severe postnatal phenotype that involved concordant hypoglycemia, growth retardation, and lethality [35]. Furthermore, liver dysfunction and damage with increased levels of aspartate aminotransferase and total bilirubin were observed in ZBTB20 null mice [35]. All these changes are opposite to the hallmarks of cancer, such as insensitivity to antigrowth signals, resisting cell death and deregulating cellular energetics. In this study, we for the first time demonstrated the oncogenic role of ZBTB20 in HCC via *in vitro* and *in vivo* experiments. Additionally, we found that the effects of ZBTB20 on HCC cells were at least partly through repression of FoxO1. Though the same mechanisms are already reported in non-small cell lung cancer [17], it is still a interesting finding that discloses the underlying molecular mechanisms involved in hepatocarcinogenesis.

## MATERIALS AND METHODS

### Clinical samples

130 HCC samples and paired normal tumor-adjacent samples (> 2 cm distance from the margin of the margin of the resection) were collected in the Department of Hepatobiliary Surgery at Nanfang Hospital, Southern Medical University Jan 2006 to Dec 2008 after obtaining informed consent. All patients did not have any other malignancies and did not receive chemotherapy or radiotherapy before operation. Specimens were frozen at −80°C for RNA and protein isolation, and were stored in paraformaldehyde for IHC assays. Clinical data were collected from the medical records and shown in Table 1. The protocols of this study were approved by the Southern Medical University Ethics Committee according to the Declaration of Helsinki (as revised in Tokyo 2004).

### Cell lines and transfection

Human HCC cell lines including Huh7, SMMC-7721, Hep3B and HepG2, and the human immortalized normal hepatocyte cell line LO2 were purchased from the Institute of Biochemistry and Cell Biology, Chinese Academy of Sciences (Shanghai, China) and maintained in Dulbecco's modified Eagle medium (DMEM, Gibco, Grand Island, NY, USA) containing 10% fetal bovine serum (FBS, Gibco) with 100 units/mL penicillin and 100 μg/mL streptomycin (Sigma, St-Louis, MO, USA) and cultured in a humidified 5% CO2 incubator at 37°C.

Retroviral vectors pMMP-ZBTB20 and pMMP-FoxO1 were generated by inserting the respective cDNA into pMMP. The retroviruses were packaged and tranfected into HCC cells as previously described [36]. The targeted sequences for ZBTB20 siRNA (5′-CUA UGC GAU UAC GAC UAA GU-3′), FoxO1 siRNA (5′-GCA AAG AUG GCC UCU ACU U-3′) and a nonspecific duplex oligonucleotide as a negative control were synthesized by Sangon Biotech (Shanghai) Co., Ltd. (Shanghai, China). Cells were transfected with the vectors mentioned above using Lipofectamine 2000 according to the manufacturer's instructions (Invitrogen, Carlsbad, CA, USA).

### Immunohistochemical staining

Formalin-fixed and paraffin-embedded specimens were cut into 4 μm thick sections, deparaffiized with dimethylbenzene, rehydrated in 100%, 95%, 90%, 80% and 75% ethanol, and quenched for endogenous peroxidase in 3% hydrogen peroxide. Then specimens were boiled in antigen-retrieval buffer containing 0.01 M sodium citrate-hydrochloric acid (pH = 6.0) for 15 min. Then the slides were incubated over night at 4°C with primary antibody against ZBTB20 (ab127702, Abcam, Cambridge, MA, USA), FoxO1 (ab39670, Abcam) or Ki-67 (#9027, Cell Signaling, Danvers, MA, USA). The biotinylated secondary antibody (ZSGB-Bio, Beijing, China) was used to detect the primary antibody. Finally, the visualization signal was developed with diaminobenzidine (DAB). The sections were counterstained with hematoxylin, dehydrated in alcohol and xylene, and finally mounted onto glass slides. The percentage of positive tumor cells was graded as per the following criteria: 0, less than 10%; 1, 10–30%; 2, 31–50%; 3, more than 50%. IHC scores were measured for 10 independent high magnification (400×) fields.

### Western blot

Cells and tissues were lysed on ice in RIPA lysis buffer according to manufacturer-provided instructions. The protein concentration was quantified with the BCA reagent (Rockford, IL, USA). Equal amounts of protein were divided to electrophoresis in SDS-PAGE gel and then transferred to a nitrocellulose membrane (Millipore, MA, USA). After blocking with 5% nonfat milk for 1 h, the membranes were incubated overnight at 4°C with primary antibody against ZBTB20, FoxO1, FoxO3 (ab12162, Abcam), Cyclin D1 (#2978, Cell Signaling Technology), Cyclin E (#4136, Cell Signaling Technology), p21 (#2947, Cell Signaling Technology), p27 (#3686, Cell Signaling Technology) and β-actin (sc-47778, Santa Cruz, CA, USA). Horseradish peroxidase-conjugated goat anti-mouse or anti-rabbit secondary antibodies (Bio-Rad, Hercules, CA, USA) were used at a proper dilution and detected using a Western Blotting Luminol Reagent (sc-2048; Santa Cruz).

### Cell proliferation, cell viability and cell cycle assays

For the proliferation assay, HCC cells were seeded into 96-well plates at 5000 cells per well for 24 hours and assessed using a Cell Proliferation ELISA, BrdU (5-bromodeoxyuridine) (chemiluminescent) (Roche, Indianapolis, IN, USA). The 3-(4,5-dimethylthiazol-2-yl) 2,5-diphenyl tetrazolium bromide (MTT, Roche) assay was used to assess cell viability at 24, 48, 72 hours. For colony formation assay, cells were seeded into 6-well plate and cultured for 14 days. Cells were fixed with 10% formaldehyde for 15 min and stained with 1% crystal violet for 5 min. For cell cycle analysis, cells were collected, fixed in 75% ice-cold ethanol. Cells were treated with Bovine pancreatic RNAase (2 μg/ml; Sigma) at 37°C for 30 min, followed by incubation with propidium iodide (20 μg/ml; Sigma) for 20 min. Cell cycle analysis was determined using a BD LSRII Flow Cytometry System with FACSDiva software (BD Bioscience, Franklin Lakes, USA).

### Real-time quantitative reverse transcription polymerase chain reaction (qRT-PCR)

Total RNAs were isolated from tissues or cells by TRIzol reagent (Invitrogen). And reverse transcriptions were performed by PrimeScript reverse transcriptase reagent kit (Takara, Osaka, Japan) following the manufacturer's protocols. In order to determine the transcripts of the interest genes, real-time PCR was performed using a One Step SYBR^®^ PrimeScript^™^ RT-PCR Kit II (Takara Bio, Shiga, Japan) and an ABI PRISM 7300 Sequence Detection System (Applied Biosystems, Foster City, CA, USA). The GAPDH was quantified as internal control to normalize the expression of each gene. All-in-One^™^ qPCR Primer against to ZBTB20 (HQP007077), FoxO1 (HQP005747) and GAPDH (HQP006940) were purchased from Genecopoeia (Guangzhou, China).

### Luciferase reporter assays

A region of FoxO1 promoter from −1000 bp to +10 bp was cloned and inserted into pGL3 vector (Promega, Madison, Wisconsin, USA). Cells were seeded in triplicate in 24-well plate and transfected with 100 ng promoter plasmids by Lipofectamine 2000 (Invitrogen, Carlsbad, CA), according to the manufacturer's instructions. 50 ng pRL-TK plasmids (Promega) expressing renilla luciferase was used to normalize the luciferase activities, which were measured using the Dual Luciferase Reporter Assay System (Promega).

### Chromatin immunoprecipitation (ChIP) assay

Chromatin immunoprecipitation assay kits were from Millipore (Billerica, Massachusetts, USA). Briefly, cells were treated with 1% formaldehyde for 15 minutes to cross-link the proteins and DNA. DNA was sheared to fragments at 200–1000 bp using several sonication. The chromatin was then incubated and precipitated with antibodies specific to ZBTB20 or IgG. The immunoprecipitated DNA fragments were detected by PCR and quantified by real-time PCR. The primer sequences are listed as following: region from −200 bp to −100 bp: sense 5′-GACCTACAGTTAGCGATTAG-3′, anti-sense 5′-CTAGCATAGGATCAGTTACTA-3′; region from −2000 bp to −1800 bp: sense 5′-ATTAGCAGCATAGC GGCATGGA-3′, anti-sense 5′-TTAGGACATGCAGTCT GACGCC-3′.

### *In vivo* experiments

A nude mouse xenograft model was established using 4–6 week-old female BALB/c nude mice. Mice were kept in laminar-flow cabinets under specific pathogen-free conditions, and handled according to the recommendations of the National Institutes of Health guidelines for care and use of laboratory animals. 5 × 10^6^ SMMC-7721 cells were inoculated subcutaneously into the flank of each nude mouse. The tumor volume for each mouse was determined by measuring two of its dimensions and then calculated as tumor volume = length × width × width/2. The developing tumors were observed over the next 3 weeks, and the mice were then sacrificed at the end of follow-up. All *in vivo* experiments protocols were approved by the Institutional Animal Care and Use Committee of Southern Medical University.

### Statistical analysis

All date are presented as the Mean ± SEM. The SPSS statistical package for Windows Version 13 (SPSS, Chicago, IL, USA) was used for the Pearson chi-square tests and the multi-variant Cox regression analysis. A two-tailed Student's *t* test, a Kaplan–Meier plot, a log-rank test, a Pearson's correlation coefficient analysis or an ANOVA was used to evaluate statistical significance using GraphPad Prism 5 software (GraphPad Software, Inc, San Diego, CA, USA). *P* < 0.05 was considered to be statistically significant.
